# Increased White Matter Coherence Following Three and Six Months of Medical Cannabis Treatment

**DOI:** 10.1089/can.2022.0097

**Published:** 2022-12-05

**Authors:** Mary Kathryn Dahlgren, Atilla Gonenc, Kelly A. Sagar, Rosemary T. Smith, Ashley M. Lambros, Celine El-Abboud, Staci A. Gruber

**Affiliations:** ^1^ Cognitive and Clinical Neuroimaging Core and McLean Hospital Imaging Center, Belmont, Massachusetts, USA.; ^2^ Marijuana Investigations for Neuroscientific Discovery (MIND) Program, McLean Hospital Imaging Center, Belmont, Massachusetts, USA.; ^3^ Department of Psychiatry, Harvard Medical School, Boston, Massachusetts, USA.

**Keywords:** medical cannabis, medical marijuana, diffusion tensor imaging, white matter, fractional anisotropy, mean diffusivity

## Abstract

**Background:**

Previous studies have demonstrated abnormal white matter (WM) microstructure in recreational cannabis consumers; however, the long-term impact of medical cannabis (MC) use on WM coherence is unknown. Accordingly, this study assessed the longitudinal impact of MC treatment on WM coherence. Given results from preclinical studies, we hypothesized that MC treatment would be associated with increased fractional anisotropy (FA) and reduced mean diffusivity (MD).

**Methods:**

As part of a larger, longitudinal investigation, patients interested in treating at least one medical condition with commercially available MC products of their choosing were assessed *before initiating* MC use (baseline *n*=37; female=25, male=12) and following three (*n*=31) and six (*n*=22) months of treatment. WM coherence was assessed via diffusion tensor imaging for bilateral regions of interest including the genu of the corpus callosum, anterior limb of the internal capsule, external capsule, and anterior corona radiata, as well as an occipital control region not expected to change over time.

**Results:**

In MC patients, FA values significantly increased bilaterally in several callosal regions relative to baseline following both 3 and 6 months of treatment; MD values significantly decreased in all callosal regions but only following 6 months of treatment. No significant changes in WM coherence were observed in the control region or in a pilot sample of treatment-as-usual patients (baseline *n*=14), suggesting that increased WM coherence observed in MC patients may be attributed to MC treatment as opposed to confounding factors. Interestingly, significant reductions in MD values correlated with higher cannabidiol (CBD) exposure but not Δ-9-tetrahydrocannabinol exposure.

**Conclusions:**

Overall, MC treatment was associated with increased WM coherence, which contrasts with prior research examining recreational cannabis consumers, likely related to inherent differences between recreational consumers and MC patients (e.g., product choice, age of onset). In addition, increased CBD exposure was associated with reduced MD following 6 months of treatment, extending evidence from preclinical research indicating that CBD may be neuroprotective against demyelination. However, additional research is needed to elucidate the clinical efficacy of MC treatment and the risks and benefits of long-term MC use.

## Introduction

Diffusion tensor imaging (DTI) assesses water movement to provide quantitative measurement of brain microstructure organization. Specifically, because water diffusion within axon bundles is restricted due to myelin sheathing, DTI techniques can assess directionality of water diffusion to measure white matter (WM) coherence (i.e., organization and integrity).^1,2^ Fractional anisotropy (FA) is a scalar measure (ranging from 0 to 1) of direction-dependent water diffusion along axon bundles, with higher values (i.e., closer to 1) reflecting better WM coherence. Conversely, mean diffusivity (MD) measures overall isotropic water diffusivity in all directions and is inversely related to FA, with higher values reflecting lower coherence. Importantly, decreased FA and increased MD have been associated with poorer cognitive performance and slower cognitive processing, particularly as a function of aging,^3–5^ underscoring the public health significance of examining WM coherence.

Furthermore, research suggests that chronic, heavy recreational cannabis use is associated with lower WM coherence.^6–8^ Specifically, relative to healthy controls, regular recreational cannabis users exhibit lower FA^9–11^ and higher MD^12–14^ in WM tracts including the corpus callosum; these alterations are associated with earlier onset,^9,13^ longer duration,^10,12^ and increased frequency of cannabis use.^15^ These reductions in WM coherence may be moderated by expression of enzymes that hydrolyze endocannabinoids such as fatty acid amide hydrolase^14^ and monoacylglycerol lipase.^11^ Interestingly, research assessing the impact of cannabis use on WM coherence has focused almost exclusively on recreational use, highlighting a need for research examining medical cannabis (MC) patients.

Given legalization efforts across the United States, increasing numbers of individuals are exploring MC, which has been demonstrated to be an effective adjunctive treatment for a variety of clinical indications.^16–19^ Current estimates indicate more than 5.5 million MC patients are registered in the United States,^20^ with chronic pain, anxiety, and sleep disturbances among the most commonly reported indications for use.^21,22^ Previous research has underscored the importance of differentiating between medical and recreational cannabis use. Recreational consumers' primary goal of use is to feel “high,” and they often choose products with greater levels of Δ-9-tetrahydrocannabinol (THC), the primary intoxicating constituent in cannabis.^23^ In contrast, MC patients' primary motive for use is symptom alleviation, and they often actively *avoid* feelings of intoxication.^21,22^

Although some MC patients use products primarily comprised of THC, which is associated with a number of therapeutic effects (e.g., analgesia, antiemesis, somnolence),^24–26^ MC patients frequently seek a broader variety of products with diverse cannabinoid profiles including cannabidiol (CBD), the primary nonintoxicating constituent in cannabis, as well as more varied routes of administration.^27,28^ In addition, recreational cannabis use is often initiated during adolescence or emerging adulthood when the brain is still developing, and frequent, heavy use during this period has been linked to poorer outcomes including altered patterns of brain connectivity^29,30^ and reduced WM coherence.^9,13^ In contrast, the majority of MC patients initiate use later in life,^31^ due, at least in part, to increased prevalence of chronic medical conditions related to aging.^32^

Preclinical work has suggested that CBD may confer neuroprotective effects against demyelination given its anti-inflammatory properties, which can reduce apoptosis of oligodendrocyte progenitor cells and protect myelinogenesis. Although the exact mechanism of action remains unknown, evidence suggests potential roles for CBD modulation of several receptor subtypes including G protein–coupled receptor 55 (GPR55), serotonin 1A receptor (5-HT1A), transient receptor potential cation channel subfamily V member 1 (TRPV1), peroxisome proliferator–activated receptor-gamma (PPAR-γ), and potentially some activity at cannabinoid receptor subtypes (CB1 and CB2).^33–36^ Additional preclinical work has also provided some evidence that THC may be associated with neurogenesis, anti-inflammatory effects, and prevention of neurodegenerative processes in animal models of disease, as well as in older animals via CB1 receptor–mediated processes and inhibition of enzymatic hydrolysis of acetylcholine.^37^

However, only one study to date has examined the specific impact of MC use on WM coherence in humans. Houston et al recently reported increased FA and reduced MD in patients with treatment-resistant epilepsy after taking Epidiolex, a highly purified oral solution of CBD.^38^ However, Epidiolex is available only by prescription and is currently FDA approved to treat rare seizure disorders; accordingly, it is not available to most MC patients. Furthermore, single-extracted, purified CBD compounds like Epidiolex are not analogous to products commercially available to MC patients, making it difficult to generalize findings to “real-world” MC patients who report using a range of diverse products.

Given the wide variety of MC products readily available to consumers, additional research is needed to assess the long-term impact of MC use in patients using commercially available products to assess the risks and benefits of these products. In the United States, current federal regulations prohibit administration of commercially available MC products in research studies, but the impact of these products can be assessed using nonrandomized, observational study designs. In previous work, we directly assessed the longitudinal impact of MC in a sample of patients using real-world MC products and found significant improvements in clinical state, pain, quality of life, cognitive function, and changes in patterns of brain activation following 3, 6, and 12 months of MC treatment.^39–42^

The current study is an extension of this work, examining WM coherence in a subsample of patients who completed DTI. Regions of interest (ROIs) including the genu of the corpus callosum, anterior limb of the internal capsule, external capsule, and anterior corona radiata were selected for this study based on previous work demonstrating regular recreational cannabis use is associated with lower WM coherence in these ROIs.^9–14^ As extensive preclinical work has demonstrated increased WM coherence is associated with the administration of cannabinoids commonly found in MC products (particularly CBD), we hypothesized that MC patients would exhibit increased FA and decreased MD following 3 and 6 months of MC treatment.

## Materials and Methods

### Patients and study design

As part of an ongoing, longitudinal study, patients interested in using MC to treat at least one medical or psychiatric indication (e.g., chronic pain, anxiety, mood, sleep) but who had not yet begun MC treatment were recruited from the New England area through several sources (e.g., online advertisements, MC certification centers). A separate pilot group of treatment-as-usual (TAU) patients with similar demographics as MC patients but who chose *not* to initiate MC treatment were also recruited.

To qualify for inclusion, all patients had to be 18 years or older. To comply with U.S. laws, MC patients were required to have either (1) a valid MC recommendation or certification card for their state, which grants access and the ability to purchase a broad variety of MC products from medical dispensaries; or (2) a plan to use widely available hemp-derived products, which are defined as containing ≤0.3% THC and do not currently require certification in the United States. Study staff did not facilitate MC certification.

To minimize the effects of previous cannabis exposure, all patients were required to be either cannabis naive (<15 lifetime uses) or abstinent from regular use (>1×/month) for at least 1 year before baseline assessments. All patients provided urine samples at each visit, which were assessed using a 12 panel CLIA Waived drug assay (Carlsbad, CA), and were required to test negative for THC metabolites at baseline and 11 other potential drugs of abuse at every visit. Contraindications for neuroimaging were exclusionary for this sample.

This study was approved by the Mass General Brigham Institutional Review Board and carried out in accordance with the Declaration of Helsinki. After receiving a complete description of study procedures, patients provided written informed consent to voluntarily participate in this study. No serious adverse events or major protocol deviations were reported during this study. Data were acquired between September 12, 2014 and February 24, 2020. It is of note that the COVID-19 pandemic and subsequent extended prohibition of in-person visits for research studies resulted in significant disruption of this longitudinal study and impacted the final sample size of the current analyses.

### Recording and quantification of MC use

Total MC uses per week were calculated from paper and pencil drug diaries, which were corroborated during monthly phone check-ins and in-person visits using a modified version of the Timeline Followback,^43^ designed to quantify use of cannabis and cannabinoid-containing products. Specifically, drug diaries included comprehensive information regarding details for each reported MC product used including mode, duration, frequency, and amount of use. Cannabinoid constituent information for each MC product was gathered from manufacturers' certificates of analysis and product labels. Patients supplied samples of their commonly used products for ultra-performance convergence chromatography at ProVerde Laboratories (Milford, MA) to confirm label information.

To control for heterogeneity of MC treatment regimens and varied potency across products, we created a standard metric of exposure to individual cannabinoids using frequency, amount, and cannabinoid content for each product.^44^ Specifically, THC and CBD exposure was quantified by calculating the amount of each product used over time and multiplying by the individual cannabinoid content of the product, which was converted into milligrams to account for different product types. Summed total amounts of THC and CBD exposure (mg/week) were calculated separately for each interval between study visits for all patients with sufficient product data (3-month follow-up *n*=22; 6-month follow-up *n*=15). In addition, urine samples positive for THC metabolites at follow-up visits were sent to Quest Diagnostics (Cambridge, MA) for gas chromatography–mass spectrometry quantification reported as creatinine normalized ratios (THC/Cr; ng/mg) to account for differences in hydration and kidney function.

### DTI methods

The current study examined DTI data at baseline compared with follow-up visits following 3 and 6 months of MC treatment. DTI data were acquired at McLean Hospital (Belmont, MA) on a Siemens 3T TIM Trio using a 12-channel phased array head coil in 30 noncollinear directions and *b*-value diffusion weights of 0 and 700 s/mm^2^ (slices=53, slice thickness=2.70 mm, field of view=222 mm, repetition time=7230 msec, echo time [TE]=103 msec). Preprocessing^45^ was conducted using Functional Magnetic Resonance Imaging of the Brain Software Library^46^ and included skull stripping, motion correction, eddy correction with reorientation of the *b* matrix, and correction for echo planar imaging/susceptibility distortions.^47^ To avoid inclusion of non-WM tissues, DTI values were obtained only in voxels with FA values >0.15.

After correction of the diffusion weighted images, FA and MD values were obtained with nonlinear least squares tensor fitting as it provides accurate noise modeling. The FA maps were registered to a study-specific template constructed using a subset of 20 patients and the DTI Toolkit, an optimized tensor-based registration tool that yields better results than scalar-based registration.^48^

**FIG. 1.**
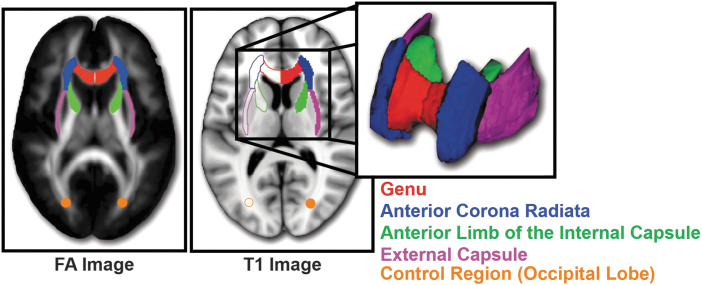
**Diffusion tensor imaging regions of interest.** Diffusion tensor imaging bilateral regions of interest identified using the Johns Hopkins University atlas, which included the genu of the corpus callosum (red), anterior limb of the internal capsule (green), external capsule (purple) and anterior corona radiata (blue). In addition, a bilateral control region was created at Montreal Neurological Institute coordinates (24, −75, 16) and (−22, −75, 16) in the occipital lobe (orange) and expanded to spheres of radius of 4 mm around those points.

Next, the John Hopkins University (JHU) atlas^49^ FA template was warped to the study-specific template using Advanced Normalization Tools^50^ and a reverse warping procedure was applied to bring the JHU labels into each individual patient's space. Images were visually inspected to ensure adequate registration. In patients' individual space, FA and MD values were calculated bilaterally focusing on several ROIs from the JHU atlas including the genu of the corpus callosum, anterior limb of the internal capsule, external capsule, and anterior corona radiata (Fig. 1). In addition, a bilateral ROI of 4 mm radius spheres was placed in the occipital lobe at Montreal Neurological Institute coordinates (24, −75, 16) and (−22, −75, 16) to serve as a control region, which was not expected to change over time.

Scanner stability was monitored over time with a 15-minute stability measurement at the beginning of each workday using the revised Functional Biomedical Informatics Research Network protocol^51^ performed on a spherical agar phantom. Signal-to-noise ratios and image ghosting were measured and compared with normative values to detect and correct out-of-spec performance.

### Statistical analyses

To assess changes in DTI variables over time, linear mixed model (LMM) analyses were conducted using SPSS version 24 for each ROI with first-order autoregressive AR(1) covariance structures (reference group=baseline DTI values). The threshold of significance (two-tailed) was Bonferroni-corrected to α=0.025 to account for bilateral assessment of all ROIs. LMM analyses compared changes in FA and MD across three timepoints (baseline, 3 months, and 6 months of MC treatment) for the MC patient group and across two timepoints (baseline and 3 months) for the pilot TAU patient group.

For ROIs with significant WM changes between baseline and follow-up, *post hoc* correlation analyses were planned to assess the relationship between WM changes and MC use variables: average MC uses/week, THC and CBD exposure, and urinary THC/Cr. For these analyses, difference scores (baseline minus 3 months and baseline minus 6 months) were calculated for the DTI variables to specifically examine changes relative to baseline. Kendall's tau (*r_τ_*) rank correlations (two-tailed) were utilized for these analyses as indicated by sample size and tests of normality.

In addition, missingness analyses of variance were performed to compare baseline assessments of patients who completed at least one follow-up assessment after 3 or 6 months with those who did not. No significant differences were observed for any baseline demographic variable; therefore, missing data were treated as missing at random.

## Results

Thirty-seven MC patients (25 female, 12 male) eligible for neuroimaging procedures completed scanning at baseline, 31 completed follow-up assessments after 3 months and 22 after 6 months of MC treatment (Table 1; flow chart in Supplementary Fig. S1). Patients reported using MC to treat a variety of medical conditions including pain (*n*=24, 64.9%), anxiety/post-traumatic stress disorder (PTSD) (*n*=19, 51.4%), sleep (*n*=16, 43.2%), mood (*n*=7, 19.0%), and attention (*n*=2, 5.4%), with most patients reporting use for one (37.8%) or two indications (40.5%). Of patients who reported previous recreational cannabis use (*n*=18), the average length of abstinence from regular use was 23.89±16.16 years.

**Table 1.** Baseline Demographic Variables for Medical Cannabis Patients and a Pilot Group of Treatment-As-Usual Patients

Demographics	MC patients (*n*=37) *n* (%) or mean±SD	TAU patients (*n*=14) *n* (%) or mean±SD	Significance (two-tailed)
Gender identity			χ^2^=4.04, *p*=0.13, ϕ=0.28
Female	25 (67.6)	11 (78.6)	
Male	12 (32.4)	2 (14.3)	
Nonbinary	0 (0.0)	1 (2.0)	
Age	47.84±17.52	39.86±15.42	*F*=2.24, *p*=0.14, η^2^=0.04
Estimated IQ (WASI)	121.68±7.60	118.57±14.50	*F*=1.00, *p*=0.32, η^2^=0.02
Race			χ^2^=6.44, *p*=0.17, ϕ=0.36
White	33 (89.2)	10 (71.4)	
Asian	2 (5.4)	3 (21.4)	
Black	2 (2.7)	0 (0.0)	
Multiracial	0 (0.0)	1 (7.1)	
Other	1 (2.7)	0 (0.0)	
Past cannabis use			χ^2^=5.13, *p*=0.08, ϕ=0.32
Cannabis naive^a^	19 (51.4)	12 (85.7)	
Past light use^b^	12 (32.4)	1 (7.1)	
Past frequent use^c^	6 (16.2)	1 (7.1)	
Cannabis abstinence (years)^d^	23.89±16.16	43.50±2.12	*F*=2.80, *p*=0.11, η^2^=0.14

^a^ Cannabis naive at baseline was defined as ≤15 lifetime uses and <1 use/month.

^b^ Past light cannabis use was defined as a previous period of ≥1 use/month and ≤2 uses/week.

^c^ Past frequent cannabis use was defined as a previous period of ≥3 uses/week.

^d^ Only patients with a history of past cannabis use reported years of abstinence.

MC, medical cannabis; TAU, treatment-as-usual; WASI, Wechsler Abbreviated Scale of Intelligence.

With regard to MC use (Table 2), across both follow-up visits, MC patients reported an average of ∼7–8 MC uses/week. Average weekly exposure to CBD was more than two times greater than average weekly exposure to THC (∼120 mg/week vs. ∼50 mg/week). Additionally, the majority of MC patients tested positive for urinary THC metabolites. Most patients reported using several different MC products (∼3/patient) with inhalation, oromucosal, and oral routes of administration most commonly reported.

**Table 2.** Medical Cannabis Use Following 3 and 6 Months of Treatment

MC use	3 Months (*n*=31)	6 Months (*n*=22)
Frequency of MC use	Mean±SD	Mean±SD
Average MC uses/week	8.31±6.83	7.24±4.62
Average MC days used/week	5.04±2.06	5.06±1.90
Average MC times used/day	1.60±0.82	1.44±0.87

Cannabinoid exposure^a^	Mean±SD	Mean±SD
Average THC mg/week	52.31±124.34	54.68±82.66
Average CBD mg/week	126.95±265.72	119.78±149.63

Urinalysis	*n* (%) or mean±SD	*n* (%) or mean±SD
Positive THC screen	15 (48.4)	15 (68.2)
THC/creatinine (ng/mg) ratio^b^	113.09±80.99	147.60±212.59

Number of MC products used	Median (IQR)	Median (IQR)
Median total MC products	3 (2.00)	3 (2.25)

Routes of administration (product type)^c^	*n* (%) or median (IQR)	*n* (%) or median (IQR)
Inhalation (e.g., flower, vape oil)	21 (67.7)	15 (68.2)
Oromucosal (e.g., sublingual oil)	14 (45.2)	9 (40.9)
Oral (e.g., edibles, capsules)	12 (38.7)	9 (40.9)
Cutaneous (e.g., cream, lotion)	2 (6.5)	1 (4.5)
Transdermal (e.g., patch)	0 (0.0)	0 (0.0)
Transmucosal (e.g., suppository)	0 (0.0)	0 (0.0)
Median total reported routes	1 (1)	1.5 (1)

^a^ Constituent information on MC products was available for *n*=22 at 3-month follow-up and *n*=15 at 6-month follow-up.

^b^ At the 3-month follow-up, one of the 15 samples that tested positive for THC metabolites was unable to be processed by Quest Diagnostics (*n*=14 for these analyses); at the 6-month follow-up, all MC patients who had a positive urine screen had further quantification analyses.

^c^ Patients could report multiple routes of administration.

CBD, cannabidiol; IQR, interquartile range; THC, Δ-9-tetrahydrocannabinol.

### DTI results

Following 3 months of MC treatment, MC patients' FA values significantly increased in the right corona radiata (*p*=0.018), with trends for increased FA in the left (*p*=0.043) and right (*p*=0.031) genu (Table 3). Following 6 months of MC treatment, MC patients' FA values significantly increased bilaterally in the left (*p*=0.001) and right (*p*=0.005) genu and unilaterally in the right (*p*=0.007) anterior limb and right external capsule (*p*=0.025), with trends for increased FA in the left (*p*=0.029) anterior limb and the right corona radiata (*p*=0.031).

**Table 3.** White Matter Coherence Following 3 and 6 Months of Medical Cannabis Treatment: Autoregressive Linear Mixed Models (Two-Tailed)

	Mixed model Main effect: visit	Baseline (*n*=37) (ref.)	3 Months (*n*=31)	6 Months (*n*=22)
	F, *p*	Mean [95% CI]	Estimate [95% CI] Significance	Estimate [95% CI] Significance
**Fractional anisotropy**				
Left genu	***F* =** **6.022, *p*=0.004**	0.549 [0.533, 0.566]	*0.013* [<*0.001, 0.026*] *t*=*2.076*, *p=0.043*, *d*=*0.348*	**0.032 [0.014, 0.050]** ***t*=3.467, *p*=0.001, *d*=0.711**
Right genu	***F* =** **4.446, *p*=0.016**	0.545 [0.527, 0.562]	*0.016* [*0.002, 0.031*] *t*=*2.213*, *p=0.031*, *d*=*0.369*	**0.030 [0.009, 0.051]** ***t*=2.887, *p*=0.005, *d*=0.653**
Left corona radiata	*F*=1.441, *p*=0.246	0.399 [0.386, 0.412]	0.007 [−0.002, 0.015] *t*=1.479, *p*=0.145, *d*=0.078	0.010 [−0.003, 0.023] *t*=1.530, *p*=0.131, *d*=0.237
Right corona radiata	*F*=*3.460*, *p*=*0.039*	0.402 [0.390, 0.415]	**0.010 [0.002, 0.018]** ***t*=2.449, *p*=0.018, *d*=0.189**	*0.013* [*0.001, 0.025*] *t*=*2.201*, *p=0.031*, *d*=*0.300*
Left external capsule	*F*=1.946, *p*=0.153	0.386 [0.378, 0.394]	0.004 [−0.002, 0.011] *t*=1.343, *p*=0.185, *d*=0.142	0.009 [<0.001, 0.018] *t*=1.948, *p*=0.056, *d*=0.391
Right external capsule	*F*=3.008, *p*=0.058	0.381 [0.371, 0.390]	0.007 [<0.001, 0.015] *t*=1.993, *p*=0.052, *d*=0.233	**0.012 [0.002, 0.023]** ***t*=2.291, *p*=0.025, *d*=0.469**
Left anterior limb	*F*=2.540, *p*=0.088	0.490 [0.478, 0.502]	0.008 [−0.003, 0.019] *t*=1.517, *p*=0.135, *d*=0.195	*0.017* [*0.002, 0.032*] *t*=*2.222*, *p=0.029*, *d*=*0.618*
Right anterior limb	***F* =** **4.012, *p*=0.024**	0.499 [0.488, 0.510]	0.005 [−0.004, 0.014] *t*=1.082, *p*=0.284, *d*=0.106	**0.018 [0.005, 0.030]** ***t*=2.777, *p*=0.007, *d*=0.656**
Left control region	*F*=0.067, *p*=0.935	0.547 [0.532, 0.561]	0.001 [−0.006, 0.008] *t*=0.275, *p*=0.785, *d*=0.098	0.002 [−0.009, 0.012] *t*=0.358, *p*=0.722, *d*=0.025
Right control region	*F*=0.186, *p*=0.830	0.531 [0.515, 0.548]	0.001 [−0.007, 0.008] *t*=0.215, *p*=0.831, *d*=0.035	−0.002 [−0.013, 0.009] *t*=0.332, *p*=0.741, *d*=0.021
**Mean diffusivity**				
Left genu	***F* =** **25.553, *p*** **< 0.001**	7.74E-4 [6.82E-4, 8.12E-4]	<0.01E-4 [−0.46E-4, 0.46E-4] *t*=0.004, *p*=0.997, *d*=0.122	**−1.89E-4 [−2.44E-4, −1.34E-4]** ***t*=6.910, *p*** **< 0.001, *d*=1.212**
Right genu	***F* =** **30.079, *p*** **< 0.001**	7.41E-4 [6.77E-4, 8.05E-4]	0.006E-4 [−0.39E-4, 0.50E-4] *t*=0.247, *p*=0.806, *d*=0.125	**−1.96E-4 [−2.49E-4, −1.42E-4]** ***t*=7.373, *p*** **< 0.001, *d*=1.267**
Left corona radiata	***F* =** **32.590, *p*** **< 0.001**	6.16E-4 [5.60E-4, 6.72E-4]	0.05E-4 [−0.29E-4, 0.38E-4] *t*=0.288, *p*=0.775, *d*=0.121	**−1.79E-4 [−2.26E-4, −1.33E-4]** ***t*=7.761, *p*** **< 0.001, *d*=1.372**
Right corona radiata	***F* =** **30.508, *p*** **< 0.001**	6.15E-4 [5.61E-4, 6.69E-4]	−0.03E-4 [−0.45E-4, 0.38E-4] *t*=0.167, *p*=0.868, *d*=0.091	**−1.75E-4 [−2.22E-4, −1.29E-4]** ***t*=7.588, *p*** **< 0.001, *d*=1.350**
Left external capsule	***F* =** **27.530, *p*** **< 0.001**	6.38E-4 [5.88E-4, 6.88E-4]	0.08E-4 [−0.41E-4, 0.58E-4] *t*=0.345, *p*=0.732, *d*=0.134	**−1.53E-4 [−2.02E-4, −1.05E-4]** ***t*=6.368, *p*** **< 0.001, *d*=1.233**
Right external capsule	***F* =** **26.639, *p*** **< 0.001**	6.31E-4 [5.81E-4, 6.81E-4]	0.06E-4 [−0.43E-4, 0.55E-4] *t*=0.247, *p*=0.806, *d*=0.133	**−1.52E-4 [−1.99E-4, −1.05E-4]** ***t*=6.537, *p*** **< 0.001, *d*=1.236**
Left anterior limb	***F* =** **25.111, *p*** **< 0.001**	6.03E-4 [5.56E-4, 6.51E-4]	−0.03E-4 [−0.56E-4, 0.50E-4] *t*=0.109, *p*=0.914, *d*=0.052	**−1.54E-4 [−2.01E-4, −1.06E-4]** ***t*=6.477, *p*** **< 0.001, *d*=1.279**
Right anterior limb	***F* =** **24.207, *p*** **< 0.001**	6.08E-4 [5.61E-4, 6.56E-4]	−0.06E-4 [−0.61E-4, 0.49E-4] *t*=0.213, *p*=0.832, *d*=0.045	**−1.51E-4 [−1.98E-4, −1.05E-4]** ***t*=6.505, *p*** **< 0.001, *d*=1.293**
Left control region	*F*=0.981, *p*=0.383	8.01E-4 [7.82E-4, 8.20E-4]	−0.07E-4 [−0.18E-4, 0.04E-4] *t*=1.308, *p*=0.198, *d*=0.024	−0.02E-4 [−0.17E-4, 0.13E-4] *t*=0.266, *p*=0.791, *d*=0.092
Right control region	*F*=0.379, *p*=0.687	8.09E-4 [7.87E-4, 8.31E-4]	−0.04E-4 [−0.17E-4, 0.09E-4] *t*=0.607, *p*=0.547, *d*=0.163	−0.08E-4 [−0.25E-4, 0.10E-4] *t*=0.871, *p*=0.387, *d*=0.227

Bold numbers are significant at Bonferroni-corrected *p*≤0.025. Italicized numbers are findings that did not survive Bonferroni correction *p*≤0.050. Significance is only noted for estimates relative to the baseline reference group.

MD values did not significantly change from baseline to 3 months of MC treatment, but significant reductions of MD values were observed bilaterally in all ROIs following 6 months of treatment (Table 3), specifically, the left and right genu, corona radiata, external capsule, and anterior limb (all *ps* < 0.001).

Importantly, no significant changes over time were observed in the control ROI for either measures of FA or MD.

In addition, to determine whether demographic variables may have impacted these results, we ran correlations examining whether age, IQ, gender, past cannabis use, and length of cannabis abstinence were associated with changes in FA and MD values from baseline to 3 months and baseline to 6 months. None of these correlations were significant (Bonferroni-corrected *p*≤0.025), suggesting no need for covariate analyses.

### Correlational analyses: changes in WM coherence versus MC use

Higher CBD exposure was associated with greater reductions of MD values following 6 months of treatment (Table 4; Supplementary Fig. S2). Significant positive correlations were observed bilaterally for all ROIs, including the left (*p*<0.001) and right (*p*=0.005) genu; left (*p*=0.013) and right (*p*=0.004) corona radiata; left (*p*=0.012) and right (*p*=0.004) external capsule; and left (*p*=0.026, trend) and right (*p*=0.020) anterior limb. No significant correlations were observed for FA within ROIs identified by the LMM analyses or for other MC use characteristics (i.e., uses/week, THC exposure, and urinary THC/Cr).

**Table 4.** Kendall's Tau Rank Correlations: Change in Mean Diffusivity Values Following 6 Months of Medical Cannabis Use Versus Medical Cannabis Characteristics (Two-Tailed)

	MC use characteristics: 6 months			
	MC uses/week (*n*=21)^a^	THC mg/week (*n*=15)^b^	CBD mg/week (*n*=15)^b^	Urinary THC/Cr (ng/mg) (*n*=12)^c^
	r_τ_ (*p*)	r_τ_ (*p*)	r_τ_ (*p*)	r_τ_ (*p*)
**Mean diffusivity difference scores (baseline – 6 months)**				
Left genu	0.169 (0.289)	−0.038 (0.843)	**0.695 (<0.001)**	0.152 (0.493)
Right genu	0.087 (0.586)	−0.153 (0.428)	**0.543 (0.005)**	<0.001 (>0.999)
Left corona radiata	0.184 (0.250)	−0.144 (0.457)	**0.478 (0.013)**	−0.046 (0.837)
Right corona radiata	0.169 (0.289)	−0.057 (0.766)	**0.562 (0.004)**	−0.015 (0.945)
Left external capsule	0.183 (0.250)	0.019 (0.921)	**0.486 (0.012)**	0.030 (0.891)
Right external capsule	0.188 (0.238)	0.029 (0.882)	**0.555 (0.004)**	<0.001 (>0.999)
Left anterior limb	0.183 (0.250)	−0.038 (0.843)	*0.429* (*0.026*)	0.061 (0.784)
Right anterior limb	0.202 (0.204)	0.019 (0.921)	**0.448 (0.020)**	0.030 (0.891)

^a^ Twenty-two MC patients completed a 6-month follow-up visit, but one patient was identified as an outlier (>3×outside the IQR) and removed from the MC uses/week analyses.

^b^ Fifteen MC patients had constituent information on MC products at the 6-month follow-up.

^c^ Fourteen MC patients tested positive for THC at their 6-month follow-up visit and had their samples sent out for urinary THC/Cr quantification, but two patients were identified as outliers (>3×outside the IQR) and removed from these analyses.

Bold numbers are significant at Bonferroni-corrected *p*≤0.025. Italicized numbers are findings that did not survive Bonferroni correction *p*≤0.050.

CBD, cannabidiol; Cr, creatinine; THC, delta-9-tetrahydrocannabinol.

### TAU pilot analyses

Fourteen TAU patients (11 female, 2 male, 1 nonbinary) completed baseline scanning, and 12 completed follow-up scanning after 3 months. TAU patients were well-matched to MC patients with no between-group differences for gender, age, IQ, and past cannabis use (Table 1). They also reported a variety of medical conditions similar to the MC group including anxiety/PTSD (*n*=13, 85.71%), pain (*n*=8, 57.14%), mood (*n*=6, 42.86%), sleep (*n*=4, 28.57%), and appetite (*n*=1, 7.14%).

LMM analyses of the DTI data assessing potential changes to WM coherence in TAU patients between baseline and the 3-month follow-up visit revealed no significant changes in either FA or MD values over time (Table 5). In addition, exploratory 2×2 LMM analyses (patient group by visit) were performed for ROIs that showed significant (or trends for significant) differences in MC patients from baseline to 3 months; these analyses demonstrated no significant main effects of group or visit or group×visit interactions (Supplementary Table S1).

**Table 5.** Pilot Data of White Matter Coherence Following 3 Months of Treatment-As-Usual: Autoregressive Linear Mixed Models (Two-Tailed)

	Mixed model Main effect: visit	Baseline (*n*=14) (ref.)	3 Months (*n*=12)
	F, *p*	Mean [95% CI]	Estimate [95% CI] Significance
**Fractional anisotropy**			
Left genu	*F*=1.847, *p*=0.201	0.532 [0.499, 0.564]	0.007 [−0.005, 0.019] *t*=1.359, *p*=0.201, *d*=0.282
Right genu	*F*=2.635, *p*=0.133	0.529 [0.499, 0.559]	0.008 [−0.003, 0.020] *t*=1.623, *p*=0.133, *d*=0.292
Left corona radiata	*F*=0.967, *p*=0.345	0.394 [0.371, 0.416]	0.009 [−0.011, 0.028] *t*=0.984, *p*=0.345, *d*=0.292
Right corona radiata	*F*=0.202, *p*=0.662	0.392 [0.368, 0.417]	0.004 [−0.016, 0.024] *t*=0.449, *p*=0.662, *d*=0.239
Left external capsule	*F*=0.003, *p*=0.954	0.366 [0.343, 0.388]	<0.001 [−0.017, 0.018] *t*=0.059, *p*=0.954, *d*=0.016
Right external capsule	*F*=1.061, *p*=0.325	0.336 [0.291, 0.381]	−0.006 [−0.020, 0.007] *t*=1.030, *p*=0.325, *d*=0.059
Left anterior limb	*F*=3.100, *p*=0.106	0.463 [0.430, 0.496]	0.020 [−0.005, 0.045] *t*=1.761, *p*=0.106, *d*=0.497
Right anterior limb	*F*=0.135, *p*=0.720	0.478 [0.449, 0.508]	−0.004 [−0.027, 0.019] *t*=0.368, *p*=0.720, *d*=0.045
Left control region	*F*=3.406, *p*=0.092	0.556 [0.523, 0.589]	0.012 [−0.002, 0.025] *t*=1.845, *p*=0.092, *d*=0.256
Right control region	*F*=2.816, *p*=0.122	0.528 [0.493, 0.562]	0.007 [−0.002, 0.015] *t*=1.678, *p*=0.122, *d*=0.335
**Mean diffusivity**			
Left genu	*F*=2.977, *p*=0.119	6.35E-4 [5.07E-4, 7.63E-4]	−0.16E-4 [−0.36E-4, 0.05E-4] *t*=1.725, *p*=0.119, *d*=0.322
Right genu	*F*=0.519, *p*=0.487	6.17E-4 [5.02E-4, 7.32E-4]	−0.086E-4 [−0.33E-4, 0.17E-4] *t*=0.720, *p*=0487, *d*=0.193
Left corona radiata	*F*=0.923, *p*=0.357	4.28E-4 [3.74E-4, 4.82E-4]	−0.05E-4 [−0.16E-4, 0.06E-4] *t*=0.961, *p*=0.357, *d*=0.015
Right corona radiata	*F*=1.379, *p*=0.264	4.26E-4 [3.71E-4, 4.81E-4]	−0.06E-4 [−0.17E-4, 0.05E-4] *t*=1.174, *p*=0.264, *d*=0.025
Left external capsule	*F*=0.707, *p*=0.418	4.73E-4 [4.17E-4, 5.25E-4]	−0.06E-4 [−0.22E-4, 0.10E-4] *t*=0.841, *p*=0.418, *d*=0.021
Right external capsule	*F*=0.014, *p*=0.907	4.75E-4 [4.23E-4, 5.271E-4]	0.01E-4 [−0.15E-4, 0.17E-4] *t*=0.120, *p*=0.907, *d*=0.042
Left anterior limb	*F*=3.159, *p*=0.103	4.49E-4 [3.95E-4, 5.02E-4]	−0.21E-4 [−0.46E-4, 0.05E-4] *t*=1.777, *p*=0.103, *d*=0.205
Right anterior limb	*F*=2.205, *p*=0.166	4.31E-4 [3.79E-4, 4.82E-4]	−0.14E-4 [−0.36E-4, 0.07E-4] *t*=1.485, *p*=0.166, *d*=0.134
Left control region	*F*=2.844, *p*=0.119	7.88E-4 [7.58E-4, 8.18E-4]	−0.14E-4 [−0.32E-4, 0.04E-4] *t*=1.686, *p*=0.119, *d*=0.316
Right control region	*F*=0.050, *p*=0.828	7.89E-4 [7.56E-4, 8.22E-4]	−0.02E-4 [−0.25E-4, 0.20E-4] *t*=0.223, *p*=0.828, *d*=0.138

Bold numbers are significant at Bonferroni-corrected *p*≤0.025. Italicized numbers are findings that did not survive Bonferroni correction *p*≤0.050.

## Discussion

Study results demonstrated increased WM coherence over time in MC patients, characterized by increased FA and decreased MD. Specifically, following 3 months of MC treatment, increased FA was observed in several ROIs; after 6 months of MC treatment, increases in FA were not only sustained but also observed in additional ROIs. Decreased MD was detected in all ROIs following 6 months of treatment. Importantly, no significant changes in WM coherence were observed in the control ROI of MC patients or within any ROI for the pilot TAU patient group, suggesting that increased WM coherence observed in MC patients may be attributed to MC treatment as opposed to confounding factors.

Additionally, CBD exposure was associated with increased WM coherence; null results were observed for all other MC use characteristics. These findings are consistent with previous work demonstrating that CBD may be neuroprotective against demyelination in animal models of disease^33–36^ and is associated with increased WM coherence in patients with epilepsy.^38^ Future research is needed to elucidate the precise mechanism of action for these findings, with preclinical research suggesting potential roles for CBD-related modulation of several receptor subtypes including GPR55, 5-HT1A, TRPV1, and PPAR-γ.^33–36^

Importantly, CBD exposure reported in the current study (∼120–127 mg/week) was relatively low compared with studies utilizing single extracted, purified CBD products. For example, Houston et al observed increased WM coherence following Epidiolex treatment at doses ranging from 15 to 25 mg/kg per day.^38^ However, most MC patients in the current study reported using broad or full-spectrum products with diverse profiles of cannabinoids and other compounds. Research suggests therapeutic response can be achieved at significantly lower doses for broad or full-spectrum products compared with single-compound, purified CBD products, likely due to the entourage effect, a theory that cannabis products containing a variety of constituents (e.g., cannabinoids, terpenoids, flavonoids) may have enhanced effects as a result of the compounds working together synergistically.^52^

For example, a preclinical study by Gallily et al^53^ examining the anti-inflammatory and anti-nociceptive effects of cannabis reported a bell-shaped dose–response curve for a CBD isolate but a linear dose–response for a full-spectrum, high-CBD product (17.9% CBD, 1.1% THC, plus other cannabinoids), suggesting that the CBD isolate had more limited dose–response relative to the full-spectrum product. In addition, a meta-analysis found that patients with refractory epilepsy treated with full-spectrum, high-CBD products reported lower average dose and fewer side effects relative to those treated with CBD isolate products.^54^ Findings from the current study demonstrating increased WM coherence following relatively low CBD exposure compared with the previous Epidiolex study^38^ provide additional support for the potential entourage effect; additional research is needed to more fully investigate differences between isolates and broad or full-spectrum products.

Previous analyses from our ongoing longitudinal study demonstrated clinical improvement in MC patients following 3 months of treatment that were sustained following 6 and 12 months of treatment.^39–42^ In the current sample of MC patients, similar clinical improvements for mood, sleep, and pain were observed; TAU patients did not demonstrate these improvements (Supplementary Table S2).

Clinical improvement and increased FA were both observed following 3 months of treatment and sustained following 6 months of treatment. Notably, decreased MD was only observed following 6 months of treatment, suggesting that increased FA appears to occur relatively quickly (i.e., within the first 3 months) following MC treatment with decreased MD only observed later. These findings extend previous research demonstrating increased WM coherence following successful treatment in studies using conventional pharmacotherapies, indicating that restoration of WM may be part of a therapeutic response.^55,56^

Furthermore, although previous studies controlled for the impact of MC treatment expectancies in analyses of clinical scale data,^40,42^ physiological metrics such as WM coherence are less likely to be impacted by self-report biases including patients' expectancies. Overall, confirmation of clinical improvement using multimodal assessments bolsters support for the clinical efficacy of MC treatment.

In addition, previously published data from this longitudinal study reported notable reductions in conventional medication use (e.g., opioids) that accompanied significant clinical improvement.^39,40^ In the current analyses, the smaller sample size of the neuroimaging subgroup did not allow for direct assessment of the impact of conventional medication use or alcohol and tobacco use on WM coherence. Preliminary analyses indicate reduced conventional medication use (specifically, reductions in opioid use) in MC patients following 3 and 6 months of treatment (Supplementary Table S3). However, given the small sample size, it is important not to overstate the results of these analyses.

Although lower FA^9–11^ and higher MD^12–14^ in WM tracts are commonly observed in recreational cannabis consumers, current study findings of *increased* WM coherence in MC patients are likely related to differences in cannabis use characteristics between recreational consumers and MC patients. In particular, research suggests that earlier age of onset of recreational cannabis use is associated with poorer WM coherence.^9,13^ Given that initiation of MC treatment generally occurs later in life relative to recreational use,^31^ MC patients are typically beyond periods of neurodevelopmental vulnerability that remain a concern for young, recreational consumers.^29,30^ Further, Filbey et al demonstrated that FA may increase with initial onset of recreational cannabis use but decrease with long-term use.^57^ However, in the current study, increased FA and decreased MD were observed following 6 months of MC use, indicating longitudinal outcomes in MC patients may differ from recreational consumers.

In addition, although recreational consumers typically choose products with greater levels of THC, MC patients frequently report using a broader variety of products with diverse cannabinoid profiles and routes of administration relative to recreational consumers.^27,28^ In the current study, increased CBD exposure but not THC exposure was associated with increased WM coherence, indicating a potential differential impact of individual cannabinoids. These findings emphasize the importance of research studies examining the long-term impact of MC use on WM coherence as well as the specific effects of individual cannabinoids.

However, it is important to note that comparisons to studies of recreational consumers are limited, as most of these studies do not collect neuroimaging data before the initiation of use. In addition, most studies assessing WM in recreational consumers are cross-sectional and not longitudinal. Gaps in the current scientific literature further underscore the importance of longitudinal studies like the current investigation.

### Limitations

It is important to note that restrictions on in-person research due to the onset of the COVID-19 pandemic resulted in significant disruption of this longitudinal study, resulting in smaller than anticipated sample sizes. Our *a priori* power analyses indicated an anticipated effect size of *d*=0.875, indicating the need for at least 22 patients per group to yield 80% power (noncentrality parameter=2.90, critical *t*=2.01). Although our MC group was sufficiently powered for the statistical analyses, the TAU group was limited in size. However, we feel the findings from the TAU control group provide critical context; therefore, to avoid overstating these results, we have emphasized that they represent preliminary analyses from a pilot group of TAU patients.

The current study design of a nonrandomized, observational, longitudinal study was selected given current federal regulations, which prohibit the use of commercially available MC products in clinical trials. This study design increases ecological validity and allows for assessment of real-world MC products currently used by patients, and given public health concerns, it is imperative to determine the safety and efficacy profiles of commercially available products. Although lack of standardized dosing results in heterogeneous MC use (a problem across all observational studies assessing cannabis use), in the current study, comprehensive assessment of individual treatment regimens facilitated a standardized metric of use, including actual THC and CBD exposure. As manufacturers labels are often inaccurate,^58,59^ our calculations of THC and CBD exposure were strengthened by ultra-performance convergence chromatography verification of cannabinoid constituents for patients' most frequently used products.

Future studies should continue to examine a variety of MC products to further assess the efficacy of different cannabinoid constituents and the impact of various routes of administration. In addition, MC patients in the current study reported use for several different indications. Although these results provide an overarching view of MC treatment, future research should examine the efficacy of MC for specific conditions.

In the current study, patients were primarily White females with above average IQ, potentially limiting the generalizability of results. Furthermore, some evidence suggests that sex differences (and sex×age interactions) may significantly impact the effects of MC treatment. For example, preclinical research has demonstrated greater antinociceptive effects of THC in female rats compared with males.^60^ In addition, cytochrome P450 enzymes that are responsible for drug metabolism and clearance are significantly impacted by sex, age, and ethnicity,^61^ and research suggests sex may impact the pharmacokinetics of cannabinoids.^62^ However, examining the impact of sex differences on WM coherence following MC treatment was beyond the scope of the current study. Future research should confirm our findings of increased WM coherence following MC treatment in underserved and underrepresented patient samples as well as comprehensively assess potential sex-specific effects.

## Conclusions

Our findings demonstrate increased WM coherence following 3 and 6 months of MC treatment. Increased CBD exposure but not THC exposure was associated with reduced MD following 6 months of treatment, extending evidence from preclinical research indicating that CBD may be neuroprotective against demyelination. Interestingly, these findings differ from results observed in studies of recreational cannabis use, likely due to inherent differences between recreational consumers and MC patients (e.g., product choice, age of onset). Future investigations including clinical trials and those assessing real-world MC products are needed to more fully elucidate the clinical efficacy of MC treatment. In particular, longitudinal studies are crucial to examine the risks and benefits of long-term MC use.

## Supplementary Material

Supplemental data

Supplemental data

Supplemental data

Supplemental data

Supplemental data

## Abbreviations Used

5-HT1A: serotonin 1A receptor
CBD: cannabidiol
DTI: diffusion tensor imaging
FA: fractional anisotropy
GPR55: G protein–coupled receptor 55
JHU: John Hopkins University
LMM: linear mixed model
MC: medical cannabis
MD: mean diffusivity
PPAR-γ: peroxisome proliferator–activated receptor-gamma
PTSD: post-traumatic stress disorder
ROIs: regions of interest
TAU: treatment-as-usual
THC: Δ-9-tetrahydrocannabinol
TRPV1: transient receptor potential cation channel subfamily V member 1
WM: white matter
