# Supplementary material for: Increased White Matter Coherence Following Three and Six Months of Medical Cannabis Treatment
Source: Cannabis Cannabinoid Res. 2022 Dec 5;7(6):827–39. doi: 10.1089/can.2022.0097 (PMC9784607; doi:10.1089/can.2022.0097)
Supplement: Supplemental data [file Supp_FigS1.docx]

**
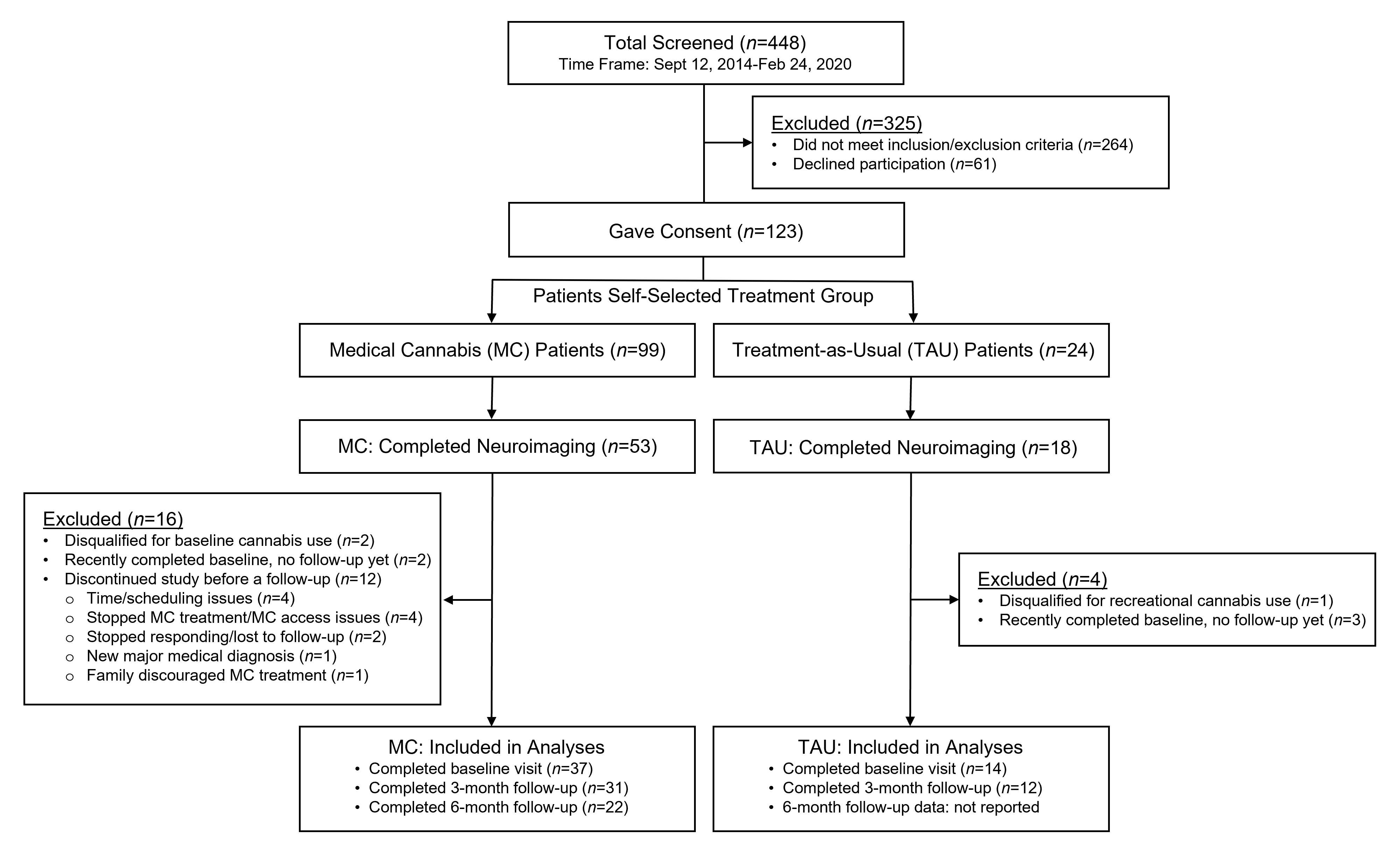
**

**Supplemental Figure 1. Study Recruitment and Enrollment Flow Chart.** Flow chart of recruitment and enrollment information for the current study, which is a subgroup analysis of diffusion tensor imaging (DTI) data from a larger, nonrandomized, longitudinal study of medical cannabis (MC) and treatment-as-usual (TAU) patients.
