# Supplementary material for: Increased White Matter Coherence Following Three and Six Months of Medical Cannabis Treatment
Source: Cannabis Cannabinoid Res. 2022 Dec 5;7(6):827–39. doi: 10.1089/can.2022.0097 (PMC9784607; doi:10.1089/can.2022.0097)
Supplement: Supplemental data [file Supp_FigS2.docx]

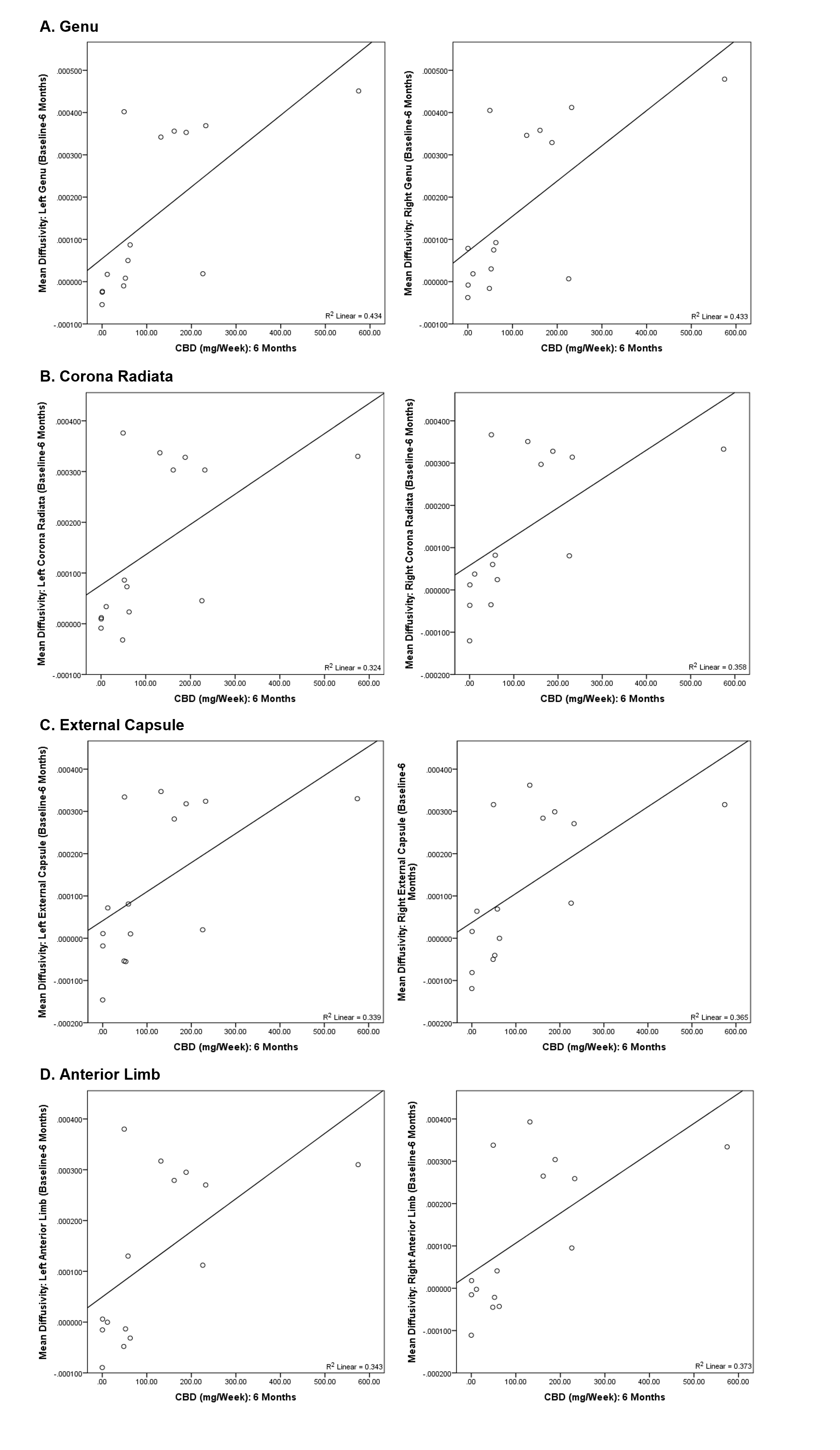


**Supplemental Figure 2. Scatterplot Graphs of the Correlation between Cannabidiol Use and Reduced Mean Diffusivity Following 6 Months of Medical Cannabis Treatment.** Scatterplot graphs demonstrating significant positive correlations between greater cannabidiol (CBD) use (average mg/week) and greater reductions in mean diffusivity (MD) bilaterally in the A) genu, B) anterior corona radiata, C) external capsule, and D) anterior limb of the internal capsule following 6 months of medical cannabis treatment. Note: Larger difference scores (baseline-6 months) indicate greater improvement (i.e., baseline MD values were much higher than MD values following 6 months of treatment with medical cannabis).
