# Supplementary material for: Increased White Matter Coherence Following Three and Six Months of Medical Cannabis Treatment
Source: Cannabis Cannabinoid Res. 2022 Dec 5;7(6):827–39. doi: 10.1089/can.2022.0097 (PMC9784607; doi:10.1089/can.2022.0097)
Supplement: Supplemental data [file Supp_TableS1.docx]

**Supplemental Table 1.** White Matter Coherence in Medical Cannabis (MC) and Treatment-As-Usual Patients (TAU) from Baseline to 3 Months: Pilot 2x2 (Group by Visit) Autoregressive Linear Mixed Models (Two-Tailed)

|  | **Visit: Baseline** | **Visit: 3 Month** | **2x2 Linear Mixed Model Results** | |
| --- | --- | --- | --- | --- |
|  | **Mean**  **[95% CI]** | **Mean**  **[95% CI]** | **Fixed Effects** | ***F* (*p*)** |
| Fractional Anisotropy:  Left Genu | |  | Main Effect: Group | 1.717 (.196) |
| MC | 0.549  [0.532, 0.566] | 0.563  [0.546, 0.581] | Main Effect: Visit | 3.222 (.080) |
| TAU | 0.532  [0.504, 0.559] | 0.541  [0.512, 0.570] | Interaction: Group*Visit | 0.143 (.707) |
| Fractional Anisotropy:  Right Genu |  |  | Main Effect: Group | 1.484 (.229) |
| MC | 0.545  [0.527, 0.562] | 0.561  [0.543, 0.580] | Main Effect: Visit | 3.640 (.063) |
| TAU | 0.529  [0.501, 0.557] | 0.539  [0.510, 0.569] | Interaction: Group*Visit | 0.220 (.641) |
| Fractional Anisotropy:  Right Corona Radiata |  |  | Main Effect: Group | 1.151 (.289) |
| MC | 0.402  [0.389, 0.415] | 0.411  [0.398, 0.424] | Main Effect: Visit | 2.147 (.150) |
| TAU | 0.392  [0.371, 0.413] | 0.396  [0.374, 0.417] | Interaction: Group*Visit | 0.383 (.539) |

**Bold** numbers are significant at Bonferroni-corrected *p*≤.025, *Italicized* numbers are findings that did not survive Bonferroni correction *p*≤.050.

**Note:** MC sample size: Baseline=37, 3 Month=31; TAU sample size: Baseline=14, 3 Month=12

**Note:** These analyses were performed only for ROIs that showed significant (or trends for significant) differences in MC patients from Baseline to 3 Months
