# Supplementary material for: Increased White Matter Coherence Following Three and Six Months of Medical Cannabis Treatment
Source: Cannabis Cannabinoid Res. 2022 Dec 5;7(6):827–39. doi: 10.1089/can.2022.0097 (PMC9784607; doi:10.1089/can.2022.0097)
Supplement: Supplemental data [file Supp_TableS2.docx]

**Supplemental Table 2.** Clinical Change Following 3 and 6 Months of Medical Cannabis (MC) Treatment or Treatment-As-Usual (TAU): Autoregressive Linear Mixed Models (LMM; Two-Tailed)

|  | **Mixed Model**  **Main Effect: Visit** | **Baseline**  ***n*=37 (ref.)** | **3 Month**  ***n*=31** | **6 Month**  ***n*=22** |
| --- | --- | --- | --- | --- |
|  | ***F* (*p*)** | **Mean**  **[95% CI]** | **Estimate [95% CI]**  **Significance** | **Estimate [95% CI]**  **Significance** |
| **LMM: MC Patients Only** |  | *n*=37 | *n*=31 | *n*=22 |
| Profile of Mood States:  Total Mood Disturbance | **3.985 (.025)** | 25.95  [14.50, 37.40] | **-7.41 [-13.92, -0.90]**  ***t*=2.285, *p*=.027, *d*=0.174** | **-13.02 [-22.73, -3.30]**  ***t*=2.682, *p*=.009, *d*=0.219** |
| Beck Depression Inventory | **9.228 (<.001)** | 9.84  [7.35, 12.33] | **-3.92 [-5.82, 2.02]**  ***t*=4.142, *p*<.001, *d*=0.525** | **-4.49 [-7.22, -1.75]**  ***t*=3.277, *p*=.002, *d*=0.458** |
| Beck Anxiety Inventory | 1.877 (.163) | 8.51  [6.02, 11.01] | *-1.78 [-3.87, 0.31]*  *t=1.712, p=.093, d=0.279* | *-2.53 [-5.49, 0.43]*  *t=1.704, p=.093, d=0.259* |
| Pittsburgh Sleep Quality Index^a^ | **4.622 (.014)** | 8.13  [6.74, 9.51] | **-2.12 [-3.54, -0.70]**  ***t*=2.995, *p*=.004, *d*=0.435** | **-1.95 [-3.85, -0.06]**  ***t*=2.055, *p*=.044, *d*=0.327** |
| Pain Visual Analogue Scale^b^ | **8.359 (.001)** | 48.20  [38.08, 58.33] | **-18.20 [-27.37, -9.03]**  ***t*=4.089, *p*<.001, *d*=0.945** | **-13.36 [-26.54, -0.18]**  ***t*=2.057, *p*=.047, *d*=0.508** |
| **LMM: TAU Patients Only** |  | *n*=14 | *n*=12 |  |
| Profile of Mood States:  Total Mood Disturbance | 0.504 (.493) | 27.29  [5.03, 49.54] | 3.63 [-7.68, 14.94]  *t*=0.710, *p*=.493, *d*=0.046 | - |
| Beck Depression Inventory | 0.499 (.494) | 11.21  [5.97, 16.46] | -1.27 [-5.21, 2.67]  *t*=0.707, *p*=.494, *d*=0.206 | - |
| Beck Anxiety Inventory | 0.014 (.907) | 8.50  [5.01, 11.99] | 0.13 [-2.33, 2.60]  *t*=0.119, *p*=.907, *d*=0.054 | - |
| Pittsburgh Sleep Quality Index | *3.939 (.078)* | 8.50  [6.53, 10.47] | *-2.06 [-4.40, 0.28]*  *t=1.985, p=.078, d=0.654* | - |
| Pain Visual Analogue Scale^b^ | 0.012 (.915) | 56.81  [33.15, 80.48] | -1.20 [-27.65, 25.24]  *t*=0.111, *p*=.915, *d*=0.132 | **-** |
| **2x2 (Group by Visit) LMM Results** | |  |  |  |
| Profile of Mood States:  Total Mood Disturbance | - | Main Effect: Group  *F*=0.396, *p*=.532 | Main Effect: Visit  *F*=0.522, *p*=.474 | *Interaction: Group*Visit*  *F*=*3.720, p*=*.061* |
| Beck Depression Inventory | - | Main Effect: Group  *F*=1.310, *p*=.258 | **Main Effect: Visit**  ***F*=11.392, *p*=.002** | *Interaction: Group*Visit*  *F*=*3.424, p*=*.071* |
| Beck Anxiety Inventory | - | Main Effect: Group  *F*=0.233, *p*=.631 | Main Effect: Visit  *F*=0.777, *p*=.383 | *Interaction: Group*Visit*  *F*=1.097, *p*=.301 |
| Pittsburgh Sleep Quality Index^a^ | - | Main Effect: Group  *F*=0.122, *p*=.729 | **Main Effect: Visit**  ***F*=8.590, *p*=.006** | *Interaction: Group*Visit*  *F*=0.001, *p*=.971 |
| Pain Visual Analogue Scale^b^ | - | *Main Effect: Group*  *F*=*3.659, p*=*.067* | **Main Effect: Visit**  ***F*=4.526, *p*=.045** | *Interaction: Group*Visit*  *F*=*3.924, p*=*.060* |

**Bold** numbers are significant at *p*≤.050; *Italicized* numbers are findings that trend towards significance at *p*≤.100

Significance is only noted for estimates relative to the baseline reference group

^a^ Two MC Patients did not complete PSQI at Baseline (*n*=35)

^b^ Not all patients reported chronic pain: MC Patients: Baseline *n*=22, 3 Month *n*=17, 6 Month *n*=11; TAU Patients: Baseline *n*=8, 3 Month *n*=7
