# Supplementary material for: Increased White Matter Coherence Following Three and Six Months of Medical Cannabis Treatment
Source: Cannabis Cannabinoid Res. 2022 Dec 5;7(6):827–39. doi: 10.1089/can.2022.0097 (PMC9784607; doi:10.1089/can.2022.0097)
Supplement: Supplemental data [file Supp_TableS3.docx]

**Supplemental Table 3.** Assessments of Conventional Medication, Alcohol, and Nicotine Use Following 3 and 6 Months of Medical Cannabis (MC) Treatment: Autoregressive Linear Mixed Models (Two-Tailed)

|  | **Mixed Model**  **Main Effect: Visit** | | **Baseline (ref.)**  **Dose (mg/week)** | **3 Month**  **Dose (mg/week)** | | **6 Month**  **Dose (mg/week)** | |
| --- | --- | --- | --- | --- | --- | --- | --- |
|  | ***F* (*p*)** | | **Mean**  **[95% CI]**  ***n*** | **Estimate [95% CI]**  **Significance**  ***n*** | | **Estimate [95% CI]**  **Significance**  ***n*** | |
| **Medical Cannabis Patients (MC)** | | | | | | | |
| Antidepressants | 1.343 (.276) | | 592.23  [302.73, 881.73]  *n*=20 | 98.50 [-41.94, 238.94]  *t*=1.432, *p*=.162, *d*=0.068  *n*=17 | | 26.78 [-169.27, 222.82]  *t*=0.278, *p*=.783, *d*=0.074  *n*=15 | |
| Benzodiazepines | 1.204 (.362) | | 14.76  [-2.89, 32.42]  *n*=11 | -7.90 [-24.23, 8.44]  *t*=1.028, *p*=.320, *d*=0.206  *n*=10 | | -15.73 [-36.96, 5.50]  *t*=1.542, *p*=.138, *d*=0.343  *n*=8 | |
| Sedatives | *2.860 (.094)* | | 30.07  [14.58, 45.56]  *n*=11 | **-24.47 [-48.11, -0.83]**  ***t*=2.267, *p*=.044, *d*=0.929**  ***n*=9** | | *-19.80 [-43.58, 3.99]*  *t=1.714, p=.099, d=0.617*  *n=8* | |
| Opioids | **4.989 (.022)** | | 360.92  [-9.70, 731.54]  *n*=10 | **-38.43 [-66.01, -10.85]**  ***t*=2.970, *p*=.010, *d*=0.332**  ***n*=9** | | **-53.12 [-93.28, -12.96]**  ***t*=2.819, *p*=.013, *d*=0.329**  ***n*=8** | |
| Mood Stabilizers/  Anticonvulsants | 0.191 (.830) | | 3893.75  [-1363.63, 9151.13]  *n*=8 | -46.65 [-1170.53, 1077.22]  *t*=0.098, *p*=.925, *d*=0.228  *n*=5 | | 278.42 [-1393.53, 1950.36]  *t*=0.392, *p*=.706, *d*=0.280  *n*=4 | |
| Stimulants | 0.635 (.550) | | 482.55  [133.88. 831.23]  *n*=7 | -189.75 [-565.83, 186.34]  *t*=1.127, *p*=.287, *d*=0.338  *n*=7 | | -146.02 [-618.85, 326.81]  *t*=0.663, *p*=.518, *d*=0.194  *n*=5 | |
| Beta Blockers | 1.507 (.273) | | 107.92  [-26.60, 242.43]  *n*=6 | <0.01 [-72.95, 72.95]  *t*<.001, *p*>.999, *d*<.001  *n*=6 | | -60.51 [-161.84, 40.83]  *t*=1.326, *p*=.214, *d*=.662  *n*=5 | |
| Muscle Relaxants | *7.920 (.064)* | | 45.53  [-55.19, 146.24]  *n*=4 | **-15.92 [-29.36, -2.49]**  ***t*=3.791, *p*=.033, *d*=0.788**  ***n*=2** | | -7.70 [-24.58, 9.19]  *t*=1.452, *p*=.243, *d*=0.650  *n*=3 | |
| Barbiturates | - | | 4.00  *n*=1 | -4.00  *n*=1 | | -0.17  *n*=1 | |
| **Non-Prescription Medications** | | | | | | | |
| NSAIDs | 0.153 (.859) | | 1517.84  [196.57, 2839.10]  *n*=13 | -332.04 [-1675.61, 1011.53]  *t*=0.519, *p*=.610, *d*=0.071  *n*=11 | | -363.67 [-1983.63, 1256.29]  *t*=0.460, *p*=.649, *d*=0.115  *n*=11 | |
| Over-the-Counter Analgesics | 1.665 (.289) | | 2932.93  [296.37, 5569.50]  *n*=3 | -1756.37 [-4748.38, 1235.64]  *t*=1.527, *p*=.189, *d*=0.773  *n*=4 | | -2370.58 [-5541.49, 800.34]  *t*=1.763, *p*=.121, *d*=0.746  *n*=4 | |
| **Other Substance Use** | | **Baseline (ref.)**  **Score** | | | **3 Months**  **Score** | | **6 Months**  **Score** |
| Alcohol:  AUDIT Score | 2.257 (.114) | | 4.08  [2.88, 5.28]  *n*=37 | 0.50 [-0.56, 1.56]  *t*=0.938, *p*=.352, *d*=0.162  *n*=31 | | -0.81 [-2.29, 0.68]  *t*=1.081, *p*=.283, *d*=0.143  *n*=22 | |
| Nicotine:  FTND Score | 0.951 (.393) | | <0.01  [-0.10, 0.10]  *n*=37 | 0.10 [-0.06, 0.25]  *t*<.001, *p*>.999, *d*=0.266  *n*=31 | | <0.01 [-0.17, 0.17]  *t*<.001, *p*>.999, *d*=no variance  *n*=22 | |

**Bold** numbers are significant at *p*≤.050, *Italicized* numbers are trends towards significance at *p*<.100.

Significance is only noted for estimates relative to the baseline reference group.

Note: No MC patients reported taking anxiolytics or antipsychotics at baseline.

Abbreviations: Alcohol Use Disorders Identification Test (AUDIT); Fagerstrom Test for Nicotine Dependence (FTND).
